# Artifact-free fat-water separation in Dixon MRI using deep learning

**DOI:** 10.1186/s40537-022-00677-1

**Published:** 2023-01-12

**Authors:** Nicolas Basty, Marjola Thanaj, Madeleine Cule, Elena P. Sorokin, Yi Liu, E. Louise Thomas, Jimmy D. Bell, Brandon Whitcher

**Affiliations:** 1 Research Centre for Optimal Health, University of Westminster, London, UK; 2 Calico Life Sciences LLC, South San Francisco, USA

**Keywords:** Neural networks, Magnetic resonance imaging, Body composition, Artifact reduction

## Abstract

Chemical-shift encoded MRI (CSE-MRI) is a widely used technique for the study of body composition and metabolic disorders, where derived fat and water signals enable the quantification of adipose tissue and muscle. The UK Biobank is acquiring whole-body Dixon MRI (a specific implementation of CSE-MRI) for over 100,000 participants. Current processing methods associated with large whole-body volumes are time intensive and prone to artifacts during fat-water separation performed by the scanner, making quantitative analysis challenging. The most common artifacts are fat-water swaps, where the labels are inverted at the voxel level. It is common for researchers to discard swapped data (generally around 10%), which is wasteful and may lead to unintended biases. Given the large number of whole-body Dixon MRI acquisitions in the UK Biobank, thousands of swaps are expected to be present in the fat and water volumes from image reconstruction performed on the scanner. If they go undetected, errors will propagate into processes such as organ segmentation, and dilute the results in population-based analyses. There is a clear need for a robust method to accurately separate fat and water volumes in big data collections like the UK Biobank. We formulate fat-water separation as a style transfer problem, where swap-free fat and water volumes are predicted from the acquired Dixon MRI data using a conditional generative adversarial network, and introduce a new loss function for the generator model. Our method is able to predict highly accurate fat and water volumes free from artifacts in the UK Biobank. We show that our model separates fat and water volumes using either single input (in-phase only) or dual input (in-phase and opposed-phase) data, with the latter producing superior results. Our proposed method enables faster and more accurate downstream analysis of body composition from Dixon MRI in population studies by eliminating the need for visual inspection or discarding data due to fat-water swaps.

**Supplementary Information:**

The online version contains supplementary material available at 10.1186/s40537-022-00677-1.

## Introduction

The UK Biobank is one of the largest collection of medical images in the world, and its abdominal imaging protocol produces a variety of magnetic resonance imaging (MRI) datasets that focus on basic structural and metabolic measurements in the thorax, abdomen and pelvis. The UK Biobank is currently acquiring images for 100,000 participants [1], with plans to scan as many of them as possible for a second time and has also performed a separate COVID-19 study [2]. Therefore, methodologies that produce minimal data waste and that all analyses resulting from this impressive collection will reach their full potential are essential. The primary dataset in the abdominal MRI protocol is a series of six acquisitions covering the neck-to-knee area of the body using a two-point Dixon method (a specific implementation of chemical-shift encoded MRI), where two three-dimensional (3D) T1-weighted volumes are acquired resulting in voxel signal intensities that depend on the difference between magnetization in fat and water [3, 4]. We will refer to the acquisitions in the UK Biobank as two-point Dixon MRI from now on to match the terminology used in [1]. Image reconstruction, performed by the MR scanner, attempts to separate the acquired volumes into pure fat and water signals. The presence of inhomogeneities in the static magnetic field during acquisition produces errors in fat-water separation performed on the scanner. When a voxel is incorrectly labelled during fat-water separation, this is known as a fat-water swap and usually occurs in a contiguous set of voxels associated with a specific tissue type, assuming a region-growing algorithm has been used. In most studies, including the UK Biobank, the phase information is not provided to the end user making a full reconstruction using the complex-valued MRI data impossible. However, the two magnitude-only 3D T1-weighted volumes acquired from the two-point Dixon sequence are retained and provide an opportunity to apply alternative reconstruction techniques.

Over the last decade MRI has become the gold standard for body composition, particularly when measuring adipose tissue, liver and pancreatic fat content. Some of these measurements have had an enormous impact on our understanding of metabolic conditions such as type-2 diabetes and non-alcoholic fatty liver disease [5]. In addition to these measurements, the data from the UK Biobank abdominal MRI protocol covers multiple tissues and organs such as muscles, abdominal organs, bones, adipose tissue, etc., with the potential for a myriad of clinically-relevant variables. Previous work on two-point Dixon MRI from the UK Biobank has identified via visual inspection approximately 4% of the participants with at least one fat-water swap in the first 40,000 scans [6, 7] or as we have previously reported via neural-network based techniques [8] from image reconstruction performed on the scanner. Using the neural-network based method in [8] enabled a census of fat-water swaps for each of the six acquisitions, where 44% of all swaps occurred in the second series, covering the chest, while only 7% occurred in the first series (neck and shoulders), 8% occurred in the third series (abdomen) and 1% in the fourth series (pelvis) [9]. Looking at the legs separately, 30% of swaps occurred in the fifth series (upper thighs) and 11% occurred in the sixth series (lower thighs and knees). Our previous method was part of an extensive image-processing pipeline and relied on one individual model for each of the magnitude-only Dixon series. This method had an error rate of approximately 1/1000, either not identifying a fat-water swap (false negative) or inducing a swap (false positive). Using that method, we were able to correct the majority of the swaps affecting entire series, though these models were not designed to identify more complicated fat-water swaps in the data leading to the motivation for the method we propose here [8].

While some reported analyses of two-point Dixon MRI made the decision to visually identify and discard data with fat-water swaps, reducing the overall number of subjects in the study by generally around 10% [6, 7, 10–13], others have applied correction in post-processing [4, 14]. Multi-point Dixon sequences ($>2$ echoes) have been developed partly to overcome this issue for fat separation and also enable fat quantification. Algorithms to perform fat separation and quantification have been developed that utilise the magnitude-only or complex-valued data, or both [15–17]. It should be noted that complex-based fat-water separation methods are sensitive to errors in the phase information from the source images and magnitude-based methods are insensitive to phase errors but are unable to fully quantify fat fraction in tissue [18]. In this work we will focus on the challenge of accurate fat separation, not fat quantification. From an operational point of view multi-point sequences come with the cost of longer acquisition times and may not be practical in protocols where speed is of the essence, and we believe the two-point Dixon technique will continue to be heavily utilised. In the case of the UK Biobank, the implementation of the two-point Dixon is a result of a trade-off between acquisition time, image quality and anatomical coverage.

The work to date on deep learning for several multiecho processing problems including fat-water separation, shows that a variety of methodologies are successful, mostly based on the U-Net [19], using two-dimensional (2D) data. While some studies have performed slice-by-slice predictions of 3D volumes, none of those found during the literature review describe fat-water separation in fully volumetric data. In this work, we train a model based on the conditional generative adversarial network (cGAN) architecture to perform swap-free fat-water separation in two-point Dixon MRI using 3D data, taking advantage of the paired nature of the input (in-phase and opposed-phase) and output (fat and water) volumes from the image acquisition and reconstruction performed on the scanner. We formulate fat-water separation as a style transfer problem, where swap-free fat and water volumes are predicted from the paired in-phase and opposed-phase volumes. We develop a new loss function inspired by the MR physics of the Dixon technique and compare it to conventional L1 loss. Our implementation of the cGAN utilises 3D patches for the neck-to-knee acquisitions.

## Literature review

Few postprocessing methodologies have been proposed that both detect and correct fat-water swaps. Glocker et al. [20] proposed a method for automated swap correction in Dixon MRI using machine learning, where fat-water swap locations are detected in whole-body volumes and corrected by inverting the voxels (fat-to-water and water-to-fat) where a swap was detected. Our previous method for swap detection and correction on a series-by-series basis also falls into this category [8]. A variety of methods for improving fat-water separation have been developed, including methods based on region growing [21], spatial smoothing [22, 23], graph cuts [24, 25], patch-based methods [26] and the projected power method [27].

Recently, deep-learning methods based on convolutional neural networks (CNNs) have been applied to separate the in-phase and opposed-phase data from a two-point Dixon acquisition into fat and water channels in 2D: fat-water separation and parameter mapping in cardiac MRI [28] and fat-water separation in whole-body Dixon MRI by predicting the full volume slice-by-slice with the real and imaginary parts of the first echo time [29]. Two-echo input data was used for fat-water separation in [30]. In [31], the authors showed that using the complex-valued data outperformed the magnitude-only data on its own for phase-based applications and reconstructions of 2D data for three different datasets. The authors in [32] used CNNs to map parameters and estimate uncertainty for fat quantification. Another recent effort for fat-water separation in 2D data that simultaneously estimates R2* and field decay has been proposed [33]. The authors apply unsupervised training without using labels to exploit the underlying physical model, and show good agreement between models that used labels and those that did not. All the above methods were based on or closely related to the U-Net architecture [19] for 2D data. A bi-directional convolutional residual network has been shown to outperform the U-Net in multi-echo gradient recalled echo data, where the results improve when increasing the number of echo times [34]. In [35], the authors proposed a CNN model that separated fat and water using real and imaginary data from six echo times of single-slice knee and head multiecho MRI.

Generative adversarial networks (GANs) are known for their ability to generate realistic outputs and have been broadly applied in medical imaging for tasks such as data augmentation, image segmentation, image super resolution [36] or compressed sensing [37], using a variety of novel architectures that rely on adversarial training [38, 39]. GANs work by training generator and discriminator networks in an adversarial manner, where the latter can enable the use of unlabeled or unpaired data and yield high perceptive quality in the output. GANs have shortcomings which include the difficulty of assessing their quality and the possible introduction or removal of abnormalities [39, 40]. These issues make GANs potentially unreliable for use in a diagnostic setting. A popular example of a GAN is the cycleGAN [41], which allows unpaired style transfer but relies on extensive computational resources. Applications in medical imaging, where paired data is uncommon, can benefit from this method; for example, to improve generisability of CT segmentations [42]. Another example of a GAN architecture is the conditional generative adversarial network (cGAN), which has been proposed for paired image-to-image style transfer [43]. It relies on paired data and an additional L1 loss that conditions the model output to match its input. In a medical imaging context, this means the anatomy in the output is guided to match the input, which is an ideal way to control the biological accuracy of the generated output. Conditional GANs have also been broadly applied in medical imaging for tasks such as data augmentation, image reconstruction and segmentation [38, 44]. A recent study has applied the cGAN architecture, using six-channel 2D data, to perform fat-water separation in addition to predicting the field map and R2* values [45]. The authors showed that their method outperforms a standard U-Net. The work to date by the deep learning community shows that a variety of methodologies, mostly based on the U-Net and using 2D data, perform well for several multiecho processing problems including fat-water separation. When more echoes are used as input, the performance of deep learning models improves. Some authors have performed slice-by-slice predictions of 3D volumes but none of those found through the literature review have performed fat-water separation in fully volumetric data.

## Materials and methods

### Data

**Fig. 1:**
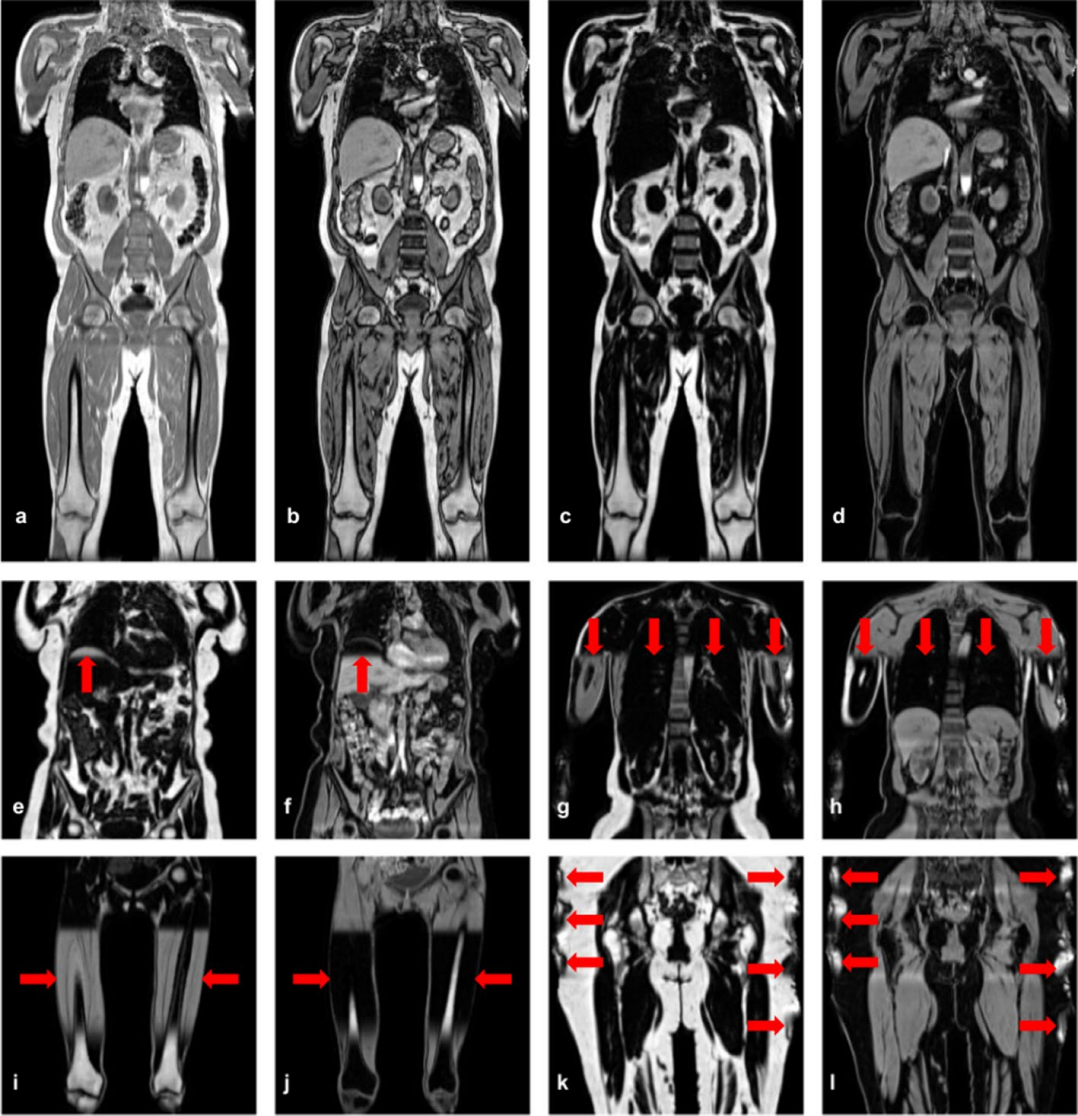
Dixon MRI data provided for representative UK Biobank participant: **a** in-phase, **b** opposed-phase, **c** fat and **d** water signals. Fat and water series with a partial swap **e**, **f** at the top of the liver, **g**, **h** a swap in a portion of the left arm and torso, **i**, **j** a swap in both legs and **k**, **l** localised swaps due to field inhomogeneities at the boundary of the field of view. Red arrows indicate fat-water swaps

The two-point Dixon technique produces two separate volumes, in-phase (*IP*) and opposed-phase (*OP*). The *IP* and *OP* volumes are acquired at times of maximum (in-phase) and minimum (opposed-phase) difference, respectively, and are used to derive water (*W*) and fat (*F*) volumes, where in theory $W=|IP+OP|/2$ and $F=|IP-OP|/2$ [3, 4]. In this study, we used the two-point Dixon MRI from the UK Biobank abdominal imaging protocol [1]. The acquisition was performed in six separate series with common parameters: TR = 6.69 ms, TE = 2.39/4.77 ms, FA = 10$^\circ$ and bandwidth = 440 Hz. All scans were performed using a Siemens Aera 1.5T scanner (Syngo MR D13) (Siemens, Erlangen, Germany). Image reconstruction on the Siemens scanner had access to the complex-valued data (both magnitude and phase) and utilised proprietary algorithms to perform online fat-water separation. The magnitude-only versions of all four volumes (*IP*, *OP*, *F*, *W*) were available for our experiments. We performed minor preprocessing to assemble the six series into a single volume for each channel [8] (Fig. 1a–d).

There are a variety of challenging fat-water swaps in the UK Biobank two-point Dixon MRI datasets, of which the most common are those affecting entire series (Fig. 1i, j). More complex swaps include partial swaps that cover only a fraction of the volume or swaps related to the multiple series acquired. For example, the top of the liver may be swapped when it appears in the second series and is isolated from all other tissue by the lungs (Fig. 1e, f), or one of the abdominal muscles may be swapped when isolated from all other tissue by internal fat (not shown). Localised fat-water swaps may also occur due to inhomogeneities at the boundary of the field of view (Fig. 1k, l). Swaps occur more frequently in subjects of extreme sizes (large and small), therefore not being able to quantify or even completely discarding these subjects may introduce bias in population studies.

For the training data, we selected 1027 participants as swap-free ground truth data for our experiments. Visual inspection of the fat and water volumes provided by the UK Biobank was performed for each participant to ensure no substantial swaps were present. Swaps, when present, only occur in the fat and water volumes, meaning the *IP* and *OP* volumes used as model input are always free of swaps. We performed this careful visual inspection to guarantee the absence of swaps in the data used as training labels, in order for the network to learn swap-free separation. The participants were chosen to cover a broad range of age, gender and body composition. During the process of quality control, we also identified more than 70 participants with at least one fat-water swap in the original *F* and *W* volumes. We used those scans to verify the performance of our technique by visual inspection. When developing the neural network model we used the *IP* and *OP* volumes as our input data and the *F* and *W* volumes as the training labels. Demographics and anthropometrics for all of the participants are provided in Table 1.

**Table 1:** Demographics and anthropometrics for participants from the UK Biobank imaging cohort used in model development and evaluation, separated by gender

	Women	Men
N	566	545
Age	62.63 ${\pm }$ 7.05 (49, 79)	63.96 ${\pm }$ 7.35 (49, 79)
Weight (kg)	66.23 ${\pm }$ 9.54 (33, 120.8)	81.95 ${\pm }$ 10.46 (57.5, 129.8)
Height (cm)	162.91 ${\pm }$ 6.12 (143, 181)	176.18 ${\pm }$ 6.79 (150, 193)
BMI (kg/$\text {m}^2$)	24.95 ${\pm }$ 3.26 (13.39, 41.8)	26.38 ${\pm }$ 2.83 (18.57, 37.93)

Values are reported as mean ${\pm }$ standard deviation and range in parentheses

### Proposed model

Given the fact that the four volumes obtained from the two-point Dixon MRI technique are paired, and the aim of this work is to produce anatomically identical voxel-to-voxel predictions, we based the models for our fat-water separation experiments on the cGAN architecture [43]. We define a generator $\mathcal {G}$ such that $\hat{F},\hat{W}=\mathcal {G}(IP,OP)$ is a direct mapping of the fat and water volumes from the in-phase (and opposed-phase) volumes, and a discriminator $\mathcal {D}$ that jointly determines whether or not the predicted volumes are truly fat and water. The objective function for the cGAN is given by

1 $$\begin{aligned} L_\text {cGAN}(\mathcal {G},\mathcal {D}) = E_{F,W}[\log \mathcal {D}({F},{W})] + E_{IP,OP}[1 - \log \mathcal {D}(\mathcal {G}(IP, OP))], \end{aligned}$$

where the loss is driven solely by the performance of the discriminator. It has been shown that additional terms in the cGAN objective function may be added to ensure the predictions are similar to the ground truth, in our case *F* and *W* were used as ground-truth labels in the L1 loss term

2 $$\begin{aligned} L_1(\mathcal {G}) = E_{F,W}[\Vert (F,W) - (\hat{F},\hat{W})\Vert _1], \end{aligned}$$

ensuring that each individual prediction matches the ground truth data. The solution

3 $$\begin{aligned} \overline{\mathcal {G}}=\arg \min _\mathcal {G}\max _\mathcal {D} L_\text {cGAN}(\mathcal {G},\mathcal {D}) + \lambda L_{1}(\mathcal {G}) \end{aligned}$$

is obtained by minimising the contribution of the generator to the objective function against a discriminator that tries to maximise it and the L1 term.

**Fig. 2:**
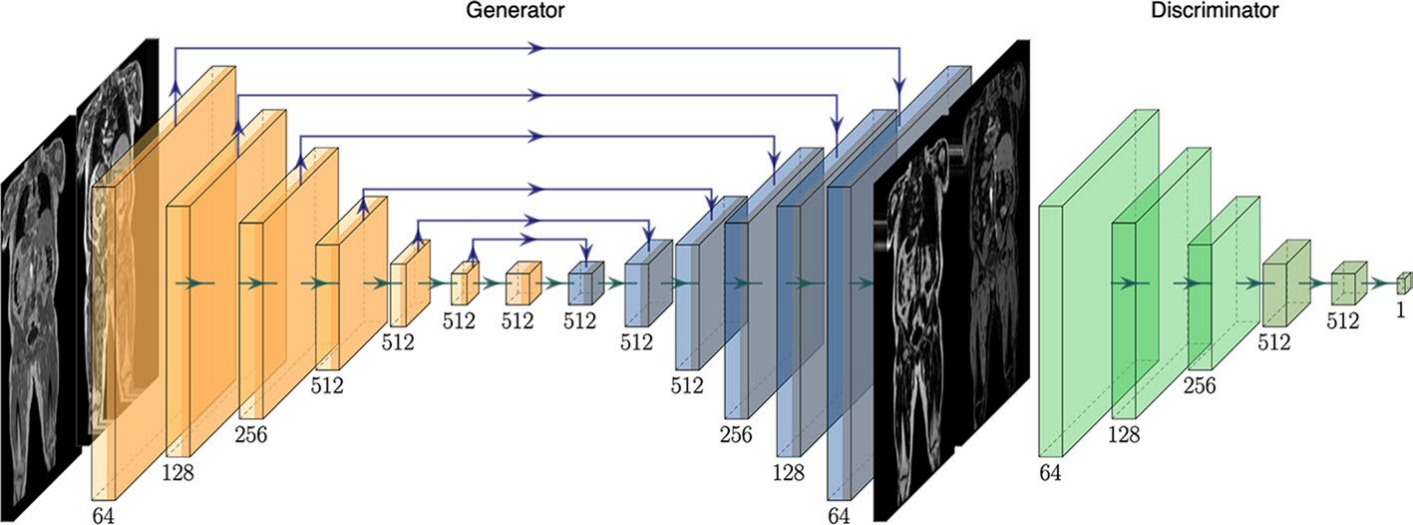
Proposed 3D cGAN architecture with *IP* and *OP* signals as input and separated $\hat{F}$ and $\hat{W}$ signals as output. Filter numbers for each convolutional block are provided. The contracting path of the generator is in orange and the expansive path is in blue. The discriminator is shown in green, the different shades indicate the change in stride length

Fat and water volumes are predicted from the input volumes, illustrated in Fig. 2. The generator follows a U-Net architecture [19] with six levels consisting of 3D convolutional layers (orange) with filter size 4 and stride 2, instead of pooling layers to move to the lower resolution levels. As the full volume dimensions were simply too large to be used as is, we set the input size to (128, 128, 128) in order to overcome memory limitations. The number of filters are indicated at each level in Fig. 2. Up-sampling (blue) is performed via 3D transpose convolutional layers with filter size 4 and stride 2. The number of filters are indicated at each level. The discriminator network follows a sequential architecture using 3D convolutional layers with filter size 4 and stride 2 (green) except the last two layers with stride 1 (dark green), to adjust the PatchGAN discriminator network to assess the volumes on a patch size of (16, 16, 16) voxels [43].

We created a data generator for the training process, which on every iteration selects a random participant and randomly crops a cube with (128, 128, 128) voxels of the input data, as well as the matching paired *W* and *F* volumes for the output ground-truth labels. The data are jointly normalized to the 99th percentile of the maximum across the intensities of all channels to have all channels on the same scale between 0 and 1, and avoid spikes in signal intensity.

We chose the number of epochs and learning rate based on an initial fine-tuning experiment with 800 participants and 200 out-of-sample data as in the validation experiments. We performed a parameter sweep from 0.0001 to 0.01, in steps of 0.01 and 0.001, and found the best results and faster convergence to be with the original cGAN implementation values of 0.0002 [43]. The hyperparameter $\lambda$ was set to 100 following the original implementation of the cGAN model in [43]. This value is similar to previous applications of the cGAN model to 3D medical imaging datasets [46, 47]. The batch size was limited by memory constraints, as we are working with large 3D arrays. We ran our experiments on a GeForce RTX 2080 Ti 12GB GPU, code in python 3.7.2, Keras 2.2.4 [48] with a tensorflow 1.13 backend. We trained the model using the Adam optimizer and batch size 2 until convergence of the generator at 100 epochs. Generator network weights of our best performing model trained on 1000 subjects, and code to process a test subject of UK Biobank Dixon MRI, will be available upon publication (https://github.com/recoh/fat_water_separation).

### Experiments

We trained three separate models with varying input data and also evaluated a new loss function. Our first experiment utilised only the *IP* volume as input data to the cGAN, a single-input model, and the swap-free *F* and *W* as ground-truth data to perform fat-water separation $IP \rightarrow \hat{F},\hat{W}$ using the L1 loss for the generator (Eq. 2) in a supervised manner. The second experiment used both the *IP* and *OP* volumes as inputs, to perform fat-water separation $IP,OP\rightarrow \hat{F},\hat{W}$ via a dual-input model. For the third experiment, we propose a new Dixon loss function that exploits the physical model describing the relationship between the derived fat and water channels from the acquired *IP* and *OP* volumes in two-point Dixon MRI. This is a similar approach followed by [49] for R2* estimation and [33], where the authors incorporated physical models normally used to estimate final parameter maps in single-slice (two-dimensional) data. These approaches remove the dependency for ground truth labels and could have significant impact on problems where labeled data are difficult to obtain or where ambiguities in the data and/or model lead to corrupted predictions, such as fat-water swaps. Similar to the second experiment, this generator model used the *IP* and *OP* as model inputs to perform the fat-water separation $IP,OP \rightarrow \hat{F},\hat{W}$. We define *IP* and *OP* error terms using the *IP* and *OP* inputs as well as the $\hat{W}$ and $\hat{F}$ generator predictions, and combine them into a Dixon loss for the generator:

4 $$\begin{aligned} L_2(\mathcal {G}) = E_{IP} [||IP - (\hat{W} + \hat{F})||_2] + E_{OP} [||OP - |\hat{W} - \hat{F}|||_2]. \end{aligned}$$

This Dixon loss function replaces the L1 loss in (3). We did not perform an equivalent fourth experiment with the Dixon loss for the single-input model generator. This is because there are obvious solutions when minimising for the in-phase data on its own $IP = \hat{W} + \hat{F}$, and no other constraints being added to training. Obvious solutions where the relationship holds would be with either the $\hat{W}$ or $\hat{F}$ data being empty, and the model output being equivalent to *IP*.

### Evaluation

To quantitatively evaluate the output of the models in our three experiments, we predicted the entire neck-to-knee $\hat{F}$ and $\hat{W}$ volumes by performing multiple patch-based predictions that cover the entire input volume. For each subject, we divided the *IP* (and *OP*) channels into twelve smaller volumes of (128, 128, 128) to match the model input size and reassembled the $\hat{F}$ and $\hat{W}$ predictions to obtain a full volume of (224, 174, 370) voxels. In voxels where multiple predicted values were produced, due to overlapping input volumes, only the first predicted value (from left-to-right, anterior-to-posterior, superior-to-inferior) was used to reassemble. Visual inspection of the predictions across the test data did not contain obvious artifacts from the patch-based algorithm. This is confirmed with the representative dataset in Additional file 2. It has been shown that using the most straightforward method for combining multiple predicted values does not sacrifice performance [50].

For all three experiments, we performed four-fold cross-validation using 800 images in a 75–25 split. We trained final models on the 800 subjects for each of the three experiments and evaluated them on the 227 subjects kept aside for testing. We used the structural similarity index measure (SSIM) [51] and peak signal-to-noise ratio (PSNR) to assess the predicted $\hat{F}$ and $\hat{W}$ volumes against the original data, as given by the fat-water separation performed on the Siemens scanner. The PSNR is given by

5 $$\begin{aligned} \text {PSNR} = 10 \cdot \log _{10}\left( \frac{\text {MPI}^2}{\text {MSE}}\right) , \end{aligned}$$

where MPI is the maximum pixel intensity and MSE is the mean squared error. SSIM is defined via

6 $$\begin{aligned} \text {SSIM}(x,y) = \frac{(2\mu _{x}\mu _{y} + C_{1})(2\sigma _{xy} + C_{2})}{(\mu _{x}^{2} + \mu _{y}^{2} + C_{1})(\sigma _{x}^{2} + \sigma _{y}^{2} + C_{2})}, \end{aligned}$$

where $\mu _x$ and $\mu _y$ represent averages of the reference image windows *x* and *y*, respectively, of the test images, $\sigma _x^2$ and $\sigma _y^2$ are the variances of the reference image windows and $\sigma _{xy}$ is their covariance. The terms $C_1$ and $C_2$ are dynamic range constants. SSIM values range from $-1$ to $+1$, the latter which is only achieved for two identical images. We computed the SSIM using the default sliding window size ($11 \times 11 \times 11)$ on the entire predicted $\hat{F}$ and $\hat{W}$ volumes.

Even though PSNR and SSIM are broadly used as the go-to metrics when it comes to assess image reconstruction quality, these metrics have drawn criticism for not necessarily corresponding to visual quality as observed by humans [52]. Since reconstructed medical images may be intended for further analyses, an assessment of the impact of the image quality on such analysis is of value, and has led to the development of semantic interpretability score (SIS) [53], which was inspired by Inception scores in GANs that assesses how well networks identify objects in generated images [54] and offers a solution of the shortcomings of GANs. The SIS may be calculated if expert manual annotations and a pre-trained segmentation model are available. The SIS is defined as the Dice overlap between segmentations generated using the reconstructed data and the manual annotations, where the scores are normalised by the average Dice score from the ground-truth images. The normalisation is performed to enable a comparison of the segmentation performance based on the reconstructed images against the baseline performance of the segmentation model on ground-truth images.

Due to the nature of swapped data, quantitative evaluation of swap correction using image quality metrics is not possible. The original data that would be used as ground truth is inherently flawed and may not be used for this purpose. For example, if our model output $\hat{F}$ and $\hat{W}$ is correct and free of swaps, but the original *F* and *W* volumes contain a swap, then the SSIM or PSNR would not be indicative of that improvement as they depend on the ground truth to be artifact-free. Similarly, the SIS score relies on an artifact-free ground truth image to compare the segmentations from the reconstructed image, which is not possible if the ground truth image is corrupted. However, the absence of swaps in our predictions may be verified via visual inspection, as well as highlighted from difference images between the original data and the model predictions. We predicted the $\hat{F}$ and $\hat{W}$ volumes for more than 70 participants that were affected by swaps identified when selecting the training datasets, and performed visual inspection on the volumes to identify whether or not the models were able to successfully perform fat-water separation where the scanner software failed.

As a final qualitative evaluation of our model on swapped data, we used the predicted fat and water channels $(\hat{F},\hat{W})$ to perform segmentations using publicly available code [8, 55] in order to highlight the importance of correct fat-water separation. We also implemented the *dixonfix* method by Glocker et al. [20] to compare our method with a closely-related alternative where the code is publicly available. In [20], the authors trained the model using 23 subjects. Acknowledging the fact that we have more training data available, we trained the *dixonfix* method using 150 (limited by computational resources) fully assembled volumes of size (224, 174, 370), a factor of 6.5 times more in terms of numbers of subjects compared with the original publication and possibly more in terms of array size. Instead of performing fat-water separation, *dixonfix* predicts binary labels of swap locations via an intermediate step of fat-water separation using a regression forest. Swap labels are then computed using a graph-cuts algorithm. The labels are used to swap the fat and water data back into the original channel.

## Results

**Fig. 3:**
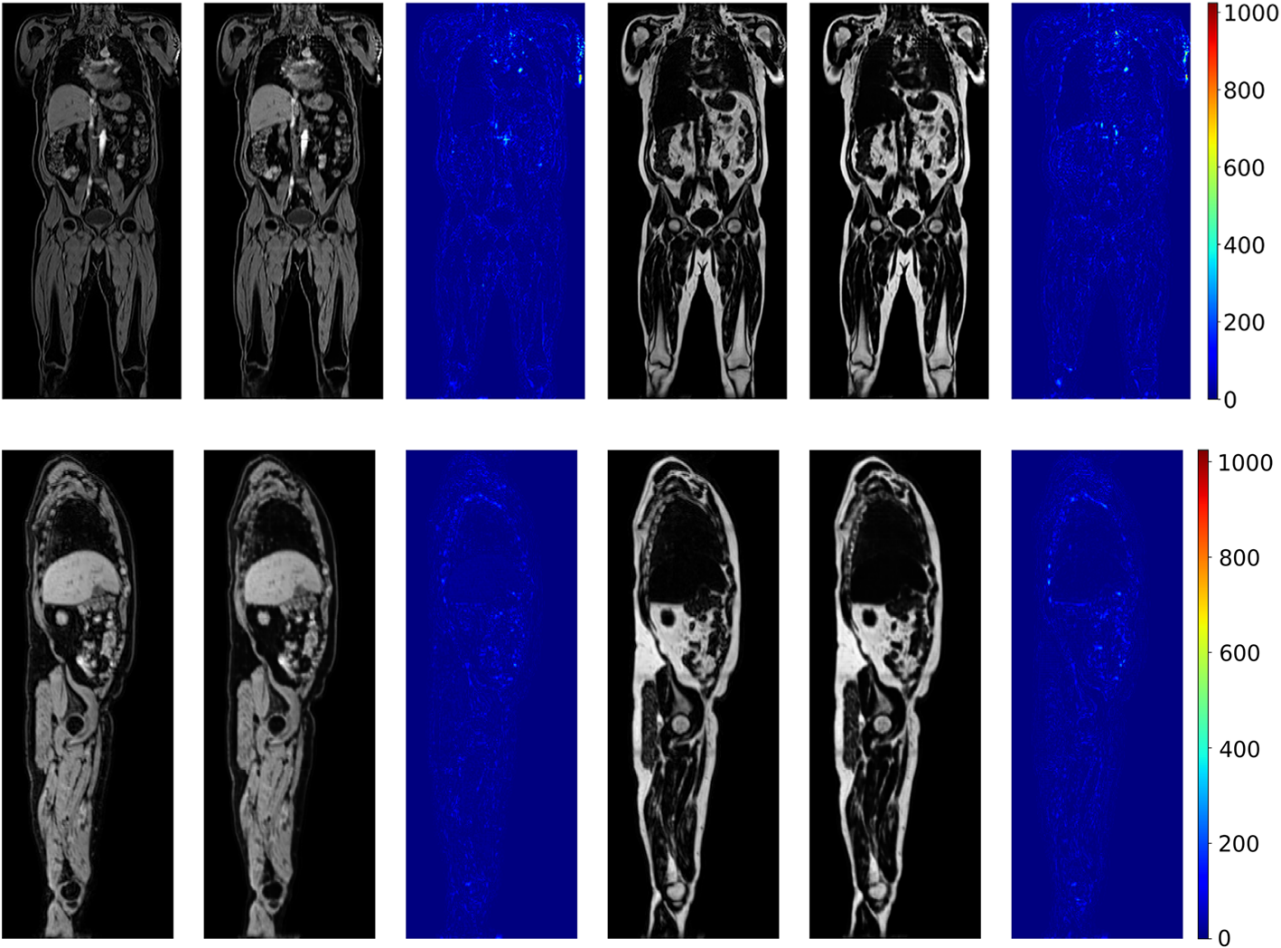
Predicted values were performed on a participant in the test set, where the model was trained on all 800 participants in the training set. Absolute differences are displayed in the original image intensities. Performance metrics calculated on the 3D volume are: $\text {SSIM}_W=0.953$, $\text {SSIM}_F=0.970$, $\text {PSNR}_W=29.80\,\text {dB}$ and $\text {PSNR}_F=29.85\,\text {dB}$

Quantitative evaluations of the three models, using four-fold cross validation on 800 participants, are provided in Table 2. The dual-input supervised model is clearly superior to the single-input supervised model, exhibiting higher SSIM and PSNR values for all runs in the cross-validation experiment. The dual-input model trained with generator Dixon loss performs better than the single-input supervised model but worse than the dual-input L1 generator model. This makes sense since the Dixon generator loss model does not benefit from ground truth labels, but has twice as much input information as the single-input model. Final models, trained on all 800 scans utilised in the cross-validation experiments and evaluated against an out-of-sample test set of 227 scans, are shown in Table 3. SSIM, PSNR and SIS values are shown in Table 4. The SIS values are based on spleen, kidney, liver and iliopsoas muscle segmentations for 65 different subjects with manual annotations. The manual annotations were performed by trained radiographers, following a standard operating procedure for each organ, and visually inspected for quality control. Representative examples may be found in Additional file 1. The SIS confirms the high level of quality from our model output, where the dual-input supervised model has the best performance across the organ segmentations. Figure 3 shows the ground-truth data, predictions and their absolute difference for the fat and water volumes of a participant in the testing set (coronal and sagittal views). An alternative version of the figure is provided in Additional files 2 and 3, where the dynamic range of the absolute differences has been reduced.

**Table 2:** Quantitative assessment of fat and water predictions compared with the original data, using four-fold cross-validation on 800 scans

Model	Run	Water		Fat	
		SSIM	PSNR (dB)	SSIM	PSNR (dB)
$IP\rightarrow \hat{F},\hat{W}$	1	0.919 ${\pm }$ 0.011	24.28 ${\pm }$ 0.78	0.945 ${\pm }$ 0.009	24.70 ${\pm }$ 0.84
	2	0.913 ${\pm }$ 0.012	24.07 ${\pm }$ 0.70	0.942 ${\pm }$ 0.008	24.45 ${\pm }$ 0.75
	3	0.926 ${\pm }$ 0.009	24.74 ${\pm }$ 0.77	0.942 ${\pm }$ 0.008	25.07 ${\pm }$ 0.83
	4	0.919 ${\pm }$ 0.010	24.35 ${\pm }$ 0.74	0.945 ${\pm }$ 0.010	24.55 ${\pm }$ 0.81
$IP,OP\rightarrow \hat{F},\hat{W}$	1	0.961 ${\pm }$ 0.006	28.99 ${\pm }$ 0.91	0.975 ${\pm }$ 0.004	29.67 ${\pm }$ 0.96
	2	0.962 ${\pm }$ 0.005	28.94 ${\pm }$ 0.82	0.972 ${\pm }$ 0.003	29.00 ${\pm }$ 0.80
	3	0.966 ${\pm }$ 0.005	29.41 ${\pm }$ 0.83	0.976 ${\pm }$ 0.004	29.58 ${\pm }$ 0.85
	4	0.963 ${\pm }$ 0.005	29.10 ${\pm }$ 0.84	0.974 ${\pm }$ 0.004	29.41 ${\pm }$ 0.83
$IP,OP\rightarrow \hat{F},\hat{W}$	1	0.930 ${\pm }$ 0.010	25.11 ${\pm }$ 0.83	0.953 ${\pm }$ 0.007	25.37 ${\pm }$ 0.91
(Dixon generator loss)	2	0.928 ${\pm }$ 0.008	25.51 ${\pm }$ 0.85	0.949 ${\pm }$ 0.007	25.51 ${\pm }$ 0.85
	3	0.935 ${\pm }$ 0.009	25.94 ${\pm }$ 0.91	0.952 ${\pm }$ 0.008	26.14 ${\pm }$ 0.96
	4	0.924 ${\pm }$ 0.009	25.36 ${\pm }$ 0.89	0.951 ${\pm }$ 0.008	25.35 ${\pm }$ 0.88

Values reported are the average and standard deviation. The three models are: single-input $IP\rightarrow \hat{F},\hat{W}$, dual-input $IP,OP\rightarrow \hat{F},\hat{W}$ and dual-input (Dixon generator loss) $IP,OP\rightarrow \hat{F},\hat{W}$

**Table 3:** Quantitative assessment of fat and water predictions compared with the original data, using all 800 scans from the cross-validation experiments for training and an out-of-sample test set of 227 scans for evaluation

Model	Water		Fat	
	SSIM	PSNR (dB)	SSIM	PSNR (dB)
$IP\rightarrow \hat{F},\hat{W}$	0.926 ${\pm }$ 0.010	24.77 ${\pm }$ 0.73	0.949 ${\pm }$ 0.008	25.22 ${\pm }$ 0.79
$IP,OP\rightarrow \hat{F},\hat{W}$	0.967 ${\pm }$ 0.005	29.47 ${\pm }$ 0.86	0.977 ${\pm }$ 0.004	29.74 ${\pm }$ 0.91
$IP,OP\rightarrow \hat{F},\hat{W}$	0.939 ${\pm }$ 0.008	26.48 ${\pm }$ 0.90	0.953 ${\pm }$ 0.007	26.73 ${\pm }$ 0.92
(Dixon generator loss)				

Values reported are the average and standard deviation

**Fig. 4:**
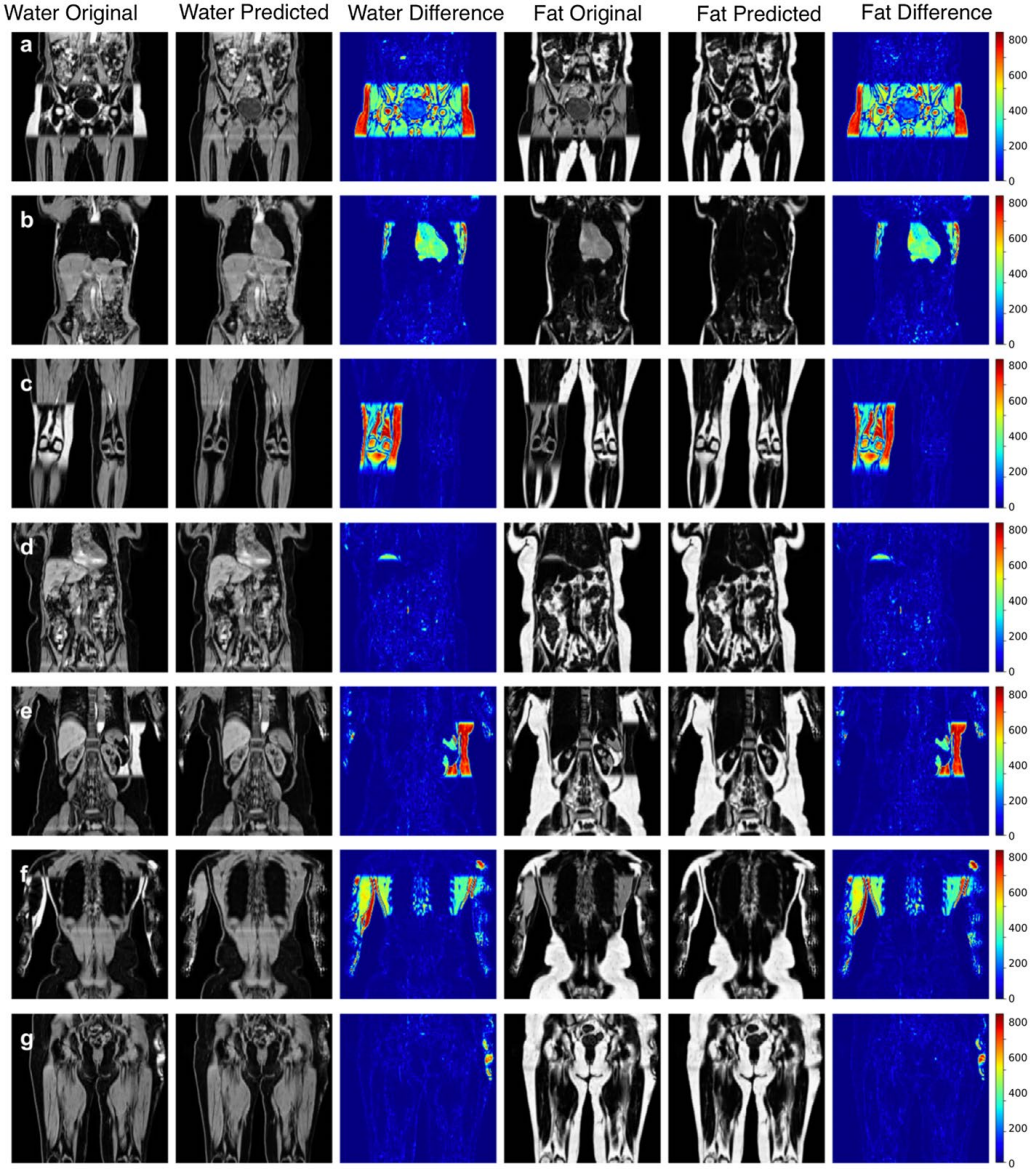
Model predictions where the original separated fat and water signals contained swaps: **a** full swap of the fourth series, **b** partial swap in second series, **c** swap in one leg, **d** swap at the top of the liver, **e** complex partial swap in the kidneys, spleen, and subcutaneous fat, **f** complex partial swap in the back and arm muscles at the edge of the field of view and **g** a partial swap at the extremities of the body due to inhomogeneities in the magnetic field at the boundary. Absolute differences are displayed in the original image intensities

Model predictions and the original data from the scanner for participants affected by various fat-water swaps are provided in Fig. 4. These examples were selected to illustrate the performance of our model in a variety of scenarios; for example, data that are not affected by major swaps, data affected by swaps that cover an entire series in the acquisition (Fig. 4a, c), data displaying complex partial (Fig. 4b, d, e) and/or boundary (Fig. 4f, g) swaps. The absolute difference images in Fig. 4 highlight where the original data have been affected by a fat-water swap in the scanner reconstruction but the model correctly predicted the fat and water signal. Visual inspection of all the other data affected by swaps showed that the model is able to perform correct fat-water separation where the scanner’s proprietary software failed.

**Fig. 5:**
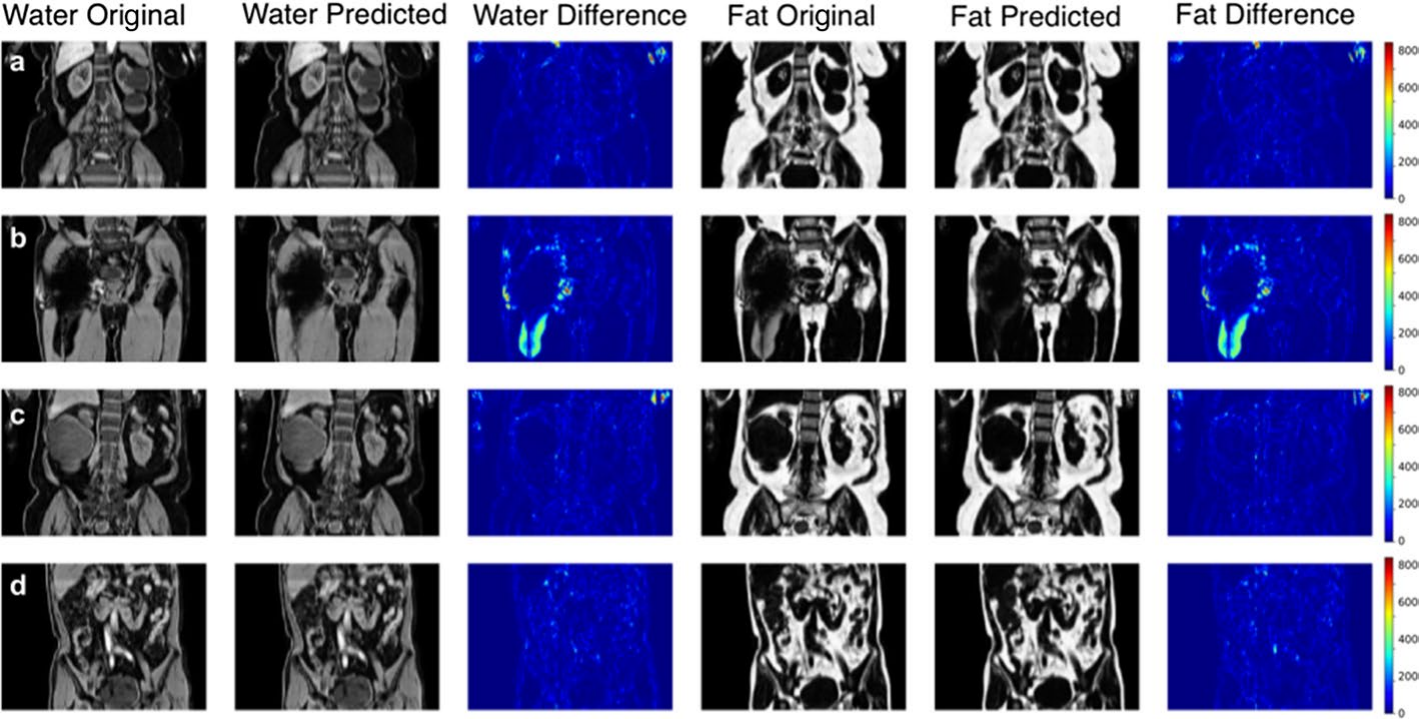
Model outputs for rare cases of various abnormalities: **a** kidney cysts, **b** hip implant and complex partial swap in the muscles, **c** large liver cyst and **d** horseshoe kidney. Absolute differences are displayed in the original image intensities

**Fig. 6:**
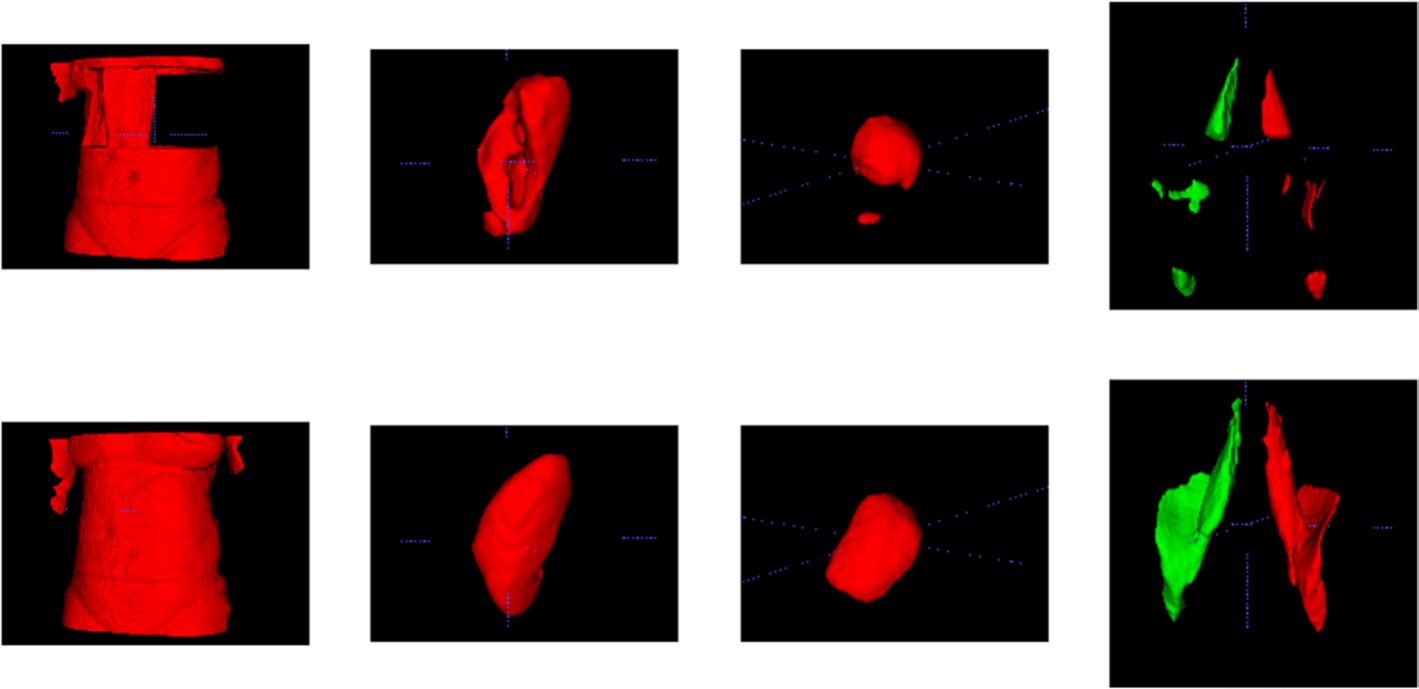
Impact of fat-water swaps on 3D segmentations. The top row shows segmentations generated using data that contained fat-water swaps and the bottom row shows segmentations of the same tissue and organs using swap-free predictions from our model. From left to right: abdominal subcutaneous fat, left kidney, spleen and iliopsoas muscles (left is red and right is green)

Figure 6 provides examples of 3D segmentations using data that suffered from fat-water swaps (top row) and the segmentation when using our model predictions (bottom row) for the following organs and tissue (from left to right): abdominal subcutaneous adipose tissue, left kidney, spleen, and left/right iliopsoas muscles (red and green, respectively). Predictions of four images with uncommon abnormalities are shown in Fig. 5. The predictions shown in Figs. 3, 4 and 5, as well as the underlying volumes used for the 3D segmentations in Fig. 6, are outputs of the final dual-input model, which performed the best across all of our experiments.

An assessment of the false positive rate between *dixonfix* and our method, using 50 out-of-sample subjects that were free of swaps, showed that our model did not induce a single false positive. Specific examples of induced fat-water swaps from the *dixonfix* method, with comparisons to our method, are provided in Additional files 4 and 5. On average, the *dixonfix* method induced 4.29 swaps inside the body, with an average misclassification of 10,803 ${\pm }$ 9400 voxels per subject, which amounts to roughly 171 ml.

## Discussion

It is common for researchers to discard two-point Dixon MRI data due to the presence of fat-water swaps in the reconstructed data produced by the scanner software. While these issues may make the data challenging, especially for non-imaging experts, we believe the two-point Dixon technique (and chemical-shift encoded MRI in general) is a powerful and efficient tool for body composition studies and therefore warrants dedicated post-processing techniques to improve fat-water separation. Our proposed model ensures that all data acquired in a study produces accurate quantitative results and that no resources, volunteers, patients, or user time are lost.

We have shown that our single- and dual-input models are able to predict swap-free fat and water data. Processing the entire neck-to-knee volumes, for example those found in the UK Biobank abdominal protocol, takes approximately eight seconds per scan. We have established the high quality of our results through quantitative metrics such as PSNR and SSIM (with average values consistently $>0.95$) for the dual-input model in both cross-validation experiments (Table 2) and out-of-sample test data on final versions of the models (Table 4). Separate quantitative evaluations, based on segmentation performance via the SIS, have highlighted the excellent performance of our models, where the dual-input model was superior. We have shown qualitative performance on scans where the scanner software failed to adequately separate the fat and water signals during reconstruction visually (Fig. 3), and by comparing to the *dixonfix* method in order to establish our method does not induce fat-water swaps (false positives) (Additional files 4 and 5). As a final qualitative validation to highlight the impact of this work, we used predicted $\hat{F}$ and $\hat{W}$ volumes as input to segmentation models and have shown (Fig. 6) how using corrected data enables meaningful segmentations. To illustrate robustness of the model, model predictions are provided for participants with a variety of abnormalities (Fig. 5). The model is able to accurately perform fat-water separation even when the input data contains structures that the model was not trained on, as shown with cysts in Fig. 5a, c, severe image artifacts such as signal dropout in Fig. 5b or rare anatomical malformations such as a horseshoe kidney in Fig. 5d. Even though the data was entirely corrupted around the hip implant in Fig. 5b, the model correctly separated the surrounding tissue where the original data contained a partial swap in the muscle. The low errors in the difference images shown in Fig. 3, as well as the regions not affected by swaps in Figs. 4 and 5, further highlight the high fidelity of the predictions to the ground truth data.

**Table 4:** Quantitative assessment of fat and water predictions compared with the original data, using the data of 65 subjects with manual annotation data

Model	Water		Fat		SIS			
	SSIM	PSNR (dB)	SSIM	PSNR (dB)	Spleen	Kidney	Liver	Iliopsoas
$IP\rightarrow \hat{F},\hat{W}$	0.927 ${\pm }$ 0.011	24.83 ${\pm }$ 1.00	0.950 ${\pm }$ 0.011	25.25 ${\pm }$ 1.12	0.858	0.802	0.965	0.935
$IP,OP\rightarrow \hat{F},\hat{W}$	0.967 ${\pm }$ 0.008	29.47 ${\pm }$ 1.57	0.976 ${\pm }$ 0.009	29.73 ${\pm }$ 1.67	0.926	0.934	0.988	0.990
$IP,OP\rightarrow \hat{F},\hat{W}$	0.941 ${\pm }$ 0.009	26.50 ${\pm }$ 1.42	0.959 ${\pm }$ 0.010	27.01 ${\pm }$ 1.56	0.895	0.898	0.965	0.959
(Dixon generator loss)								

In addition to PSNR and SSIM, we computed the semantic interpretability scores (SIS) for the spleen, kidney, liver and iliopsoas muscle segmentations

We successfully separated fat and water volumes for Dixon MRI in our three experiments, where the best model utilised both the in-phase and opposed-phase data as input with an L1 loss function. We showed that our method correctly separates *IP* and *OP* volumes and eliminates a wide variety of fat-water swaps, including those explicitly excluded from the training data (e.g., swaps that completely cover one of the series acquired, cover half the series in the legs, top-of-the-liver swaps). Minor fat-water swaps located at the boundary of the field of view or only involving the arms were included in the training data as they occur infrequently and at random anatomical locations. We hypothesise that the infrequency and randomness of these types of swaps in the training data means that the model ignores them when optimising the generator. In Fig. 4, particularly rows e-g, it appears that the model ignored and minimised the effect of the those swaps even though they were almost certainly present in the training data.

While the method is broadly applicable to two-point Dixon volumes, the particular models we trained are intended for UK Biobank Dixon MRI and may not work on data obtained using different acquisition parameters. This is a common issue encountered in highly-specialised deep learning experiments. We have benefited from the fact that the UK Biobank is a large-scale database with a fixed acquisition protocol. We anticipate that the model may be applied to similar acquisitions, such as the first two echos from a three-point Dixon scan or a two-point Dixon acquisition at a different field strength or from a different scanner manufacturer. However, caution should be exercised. Given the scanner-based fat-water reconstruction should be available, a quick application on a small number of datasets will provide the end user with constructive feedback using a quantitative performance metric like SSIM or PSNR. Given the range of the demographics and anthropometrics in the UK Biobank imaging cohort we believe that the model is applicable to other adult populations, and potentially applicable to adolescent or young-adult populations. Again, caution should be exercised since fundamental anthropometric values may be outside the ranges in the UK Biobank imaging cohort. Further investigation will need to be performed in order to assess the broader applicability on different acquisition protocols and populations.

When assessing the false positive rate of our model and comparing it to the *dixonfix* method, no false positives were detected while the latter introduced on average an error of approximately 10,000 incorrectly-swapped voxels per subject, corresponding to roughly 171 ml in volume. This could have a significant impact on downstream analyses of structures such as visceral adipose tissue, muscles and in particular smaller abdominal organs (liver, spleen, kidneys, etc). While the authors in [20] pointed out that all swaps were corrected, they did underline the fact that every prediction induced false positives in the 3D volume. These incorrectly-swapped voxels may appear in unimportant areas of the data, but we observed some induced swaps in structures such as skeletal muscle and adipose tissue. Depending on the downstream analyses performed after fat-water swap correction, such small errors may be acceptable. Further assessment on the impact of fat-water swap corrections with consistent false positives or false negatives (i.e., fat-water swaps that are ignored) in large-scale population studies is an interesting direction of investigation but outside the scope of this work.

## Conclusions and future work

We have shown that our method allows for fast and reliable fat-water separation, the best results were produced by our dual-input model. We have also shown that our method correctly separates the fat and water volumes where the scanner did not, without introducing false negatives. We have highlighted the impact of our contribution by performing segmentations of original swapped data. By doing this, we illustrate the high quality of our predictions. Given the accuracy of the predictions demonstrated here we recommend applying our methodology across all Dixon datasets in the UK Biobank imaging cohort, eliminating the need to identify acquisitions that have been corrupted by fat-water swaps. This will save the end user a substantial amount of time and resources in image processing and improve the performance of downstream quantitative analyses.

The CNNs were trained on original fat and water channels and produced good results with the model predictions, however, CNNs are known for being highly specific and not able to generalise well. While the method is broadly applicable to two-point Dixon volumes, our model is intended for UK Biobank Dixon MRI. We expect the model to perform well on similar acquisition protocols and on similar adult populations. Caution should be exercised when applying our model outside of the UK Biobank.

Future work will involve incorporating more of the preprocessing steps from our image analysis pipeline (https://github.com/recoh/pipeline) into the neural-network model. For example, the input is assumed to have bias-field correction performed but this is computationally expensive. If our model assumes the input volumes (in-phase and opposed-phase) are not bias-field corrected, but trained using the bias-field corrected fat and water volumes, then the model will learn to correct the signal intensities in addition to producing accurate fat-water separation. This has the potential to decrease processing time by an order of magnitude. It is possible that the models already perform well on such unprocessed data and is something we will assess quantitatively going forward.

The current version of our model does not operate on the full volume, due to memory constraints. We believe the process would further benefit from operating on the entire volume instead of the current patch-based implementation. Finally, we will explore possibilities of turning our third experiment, using the Dixon loss function for the generator, into a fully self-supervised framework where the discriminator will only utilise the in-phase and opposed-phase channels as ground truth for the perceptive loss, completely removing the need for the fat and water ground-truth data in training.

## Supplementary Information

**Additional file 1.** Manual annotation examples for four subjects. From left to right: iliopsoas muscles, liver, spleen, kidneys.

**Additional file 2.** Original and predicted fat and water signals and their absolute differences in coronal view with reduced dynamic range.

**Additional file 3.** Original and predicted fat and water signals and their absolute differences in sagittal view with reduced dynamic range.

**Additional file 4.** Comparative assessment of false positives between dixonfix and our method for four examples with swap-free original data. Red arrows point to false positive induced swaps.

**Additional file 5.** Comparative assessment of false positives between dixonfix and our method for four examples with original data containing swaps. Red arrows point to false positive induced swaps.

## Data Availability

Researchers may apply to use the UK Biobank data resource by submitting a health-related research proposal that is in the public interest. More information may be found on the UK Biobank researchers and resource catalogue pages (https://www.ukbiobank.ac.uk).

## Abbreviations

MRI: Magnetic resonance imaging
GAN: Generative adversarial network
cGAN: Conditional generative adversarial network
CNN: Convolutional neural network
2D: Two dimensional
3D: Three dimensional
PSNR: Peak signal-to-noise ratio
MPI: Maximum pixel intensity
MSE: Mean squared error
SSIM: Structural similarity index measure
SIS: Semantic interpretability score
