# Supplementary figures and images for: Artifact-free fat-water separation in Dixon MRI using deep learning
Source: J Big Data. 2023 Jan 12;10(1):4. doi: 10.1186/s40537-022-00677-1 (PMC9835035; doi:10.1186/s40537-022-00677-1)

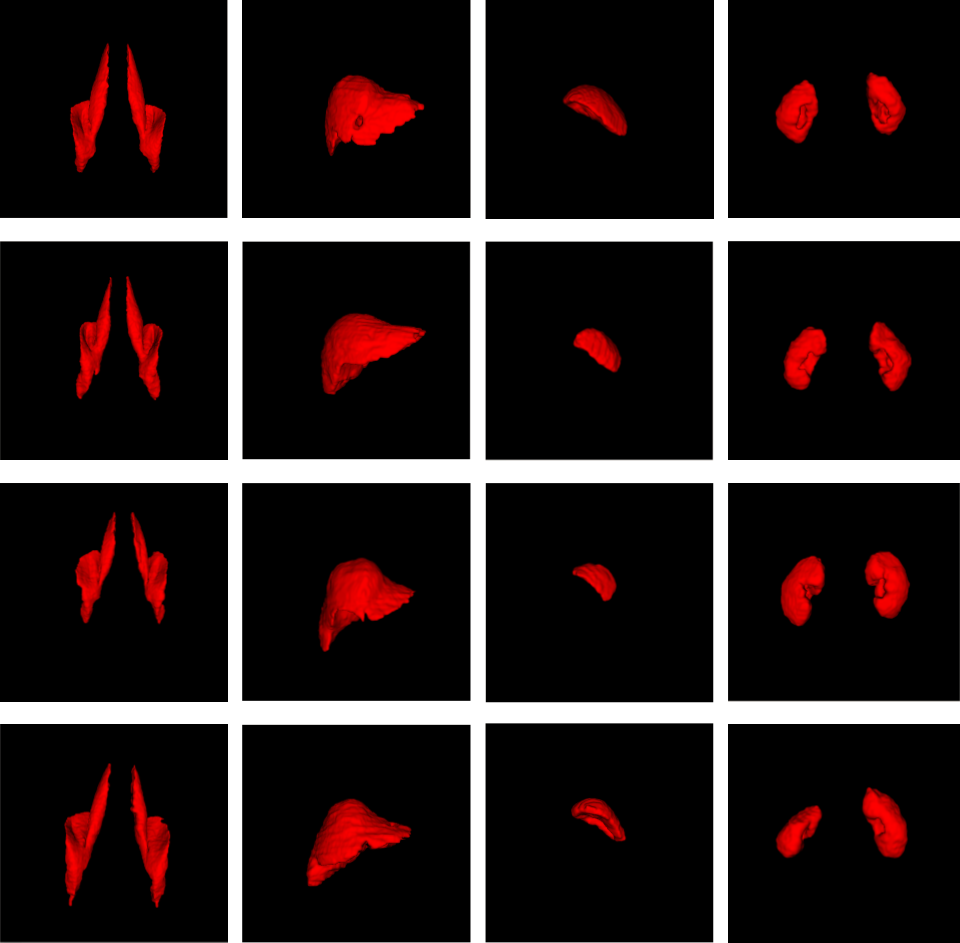

Supplement: Supplementary file 1 — Additional file 1. Manual annotation examples for four subjects. From left to right: iliopsoas muscles, liver, spleen, kidneys. [file 40537_2022_677_MOESM1_ESM.png]

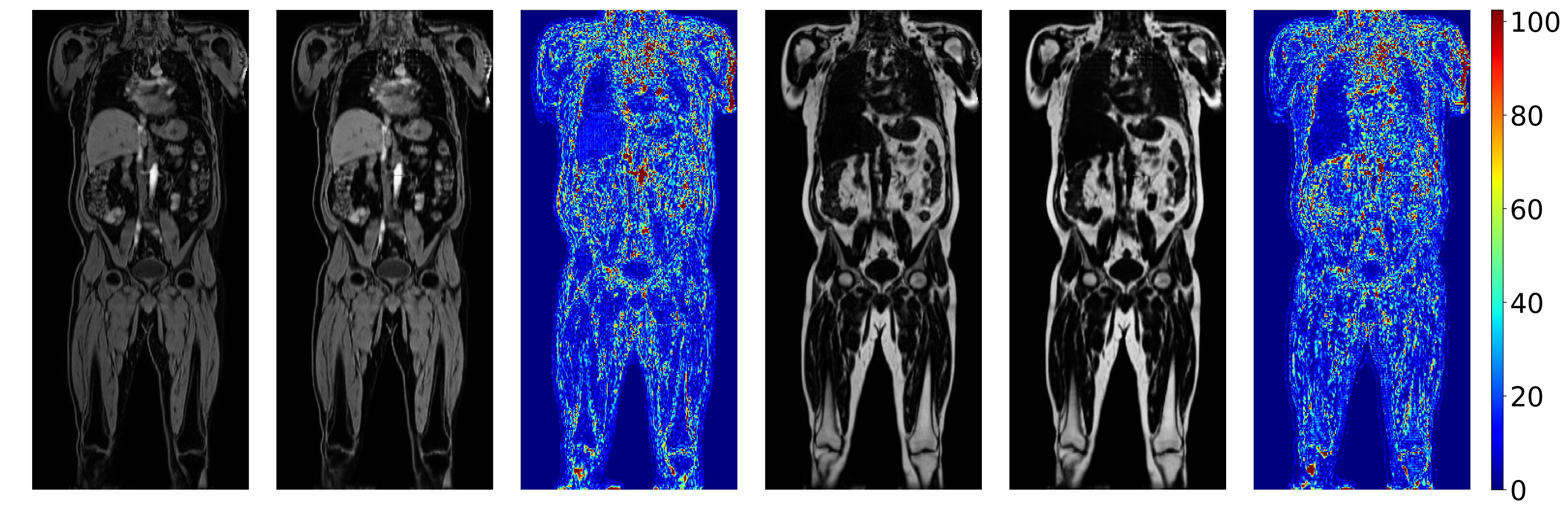

Supplement: Supplementary file 2 — Additional file 2. Original and predicted fat and water signals and their absolute differences in coronal view with reduced dynamic range. [file 40537_2022_677_MOESM2_ESM.png]

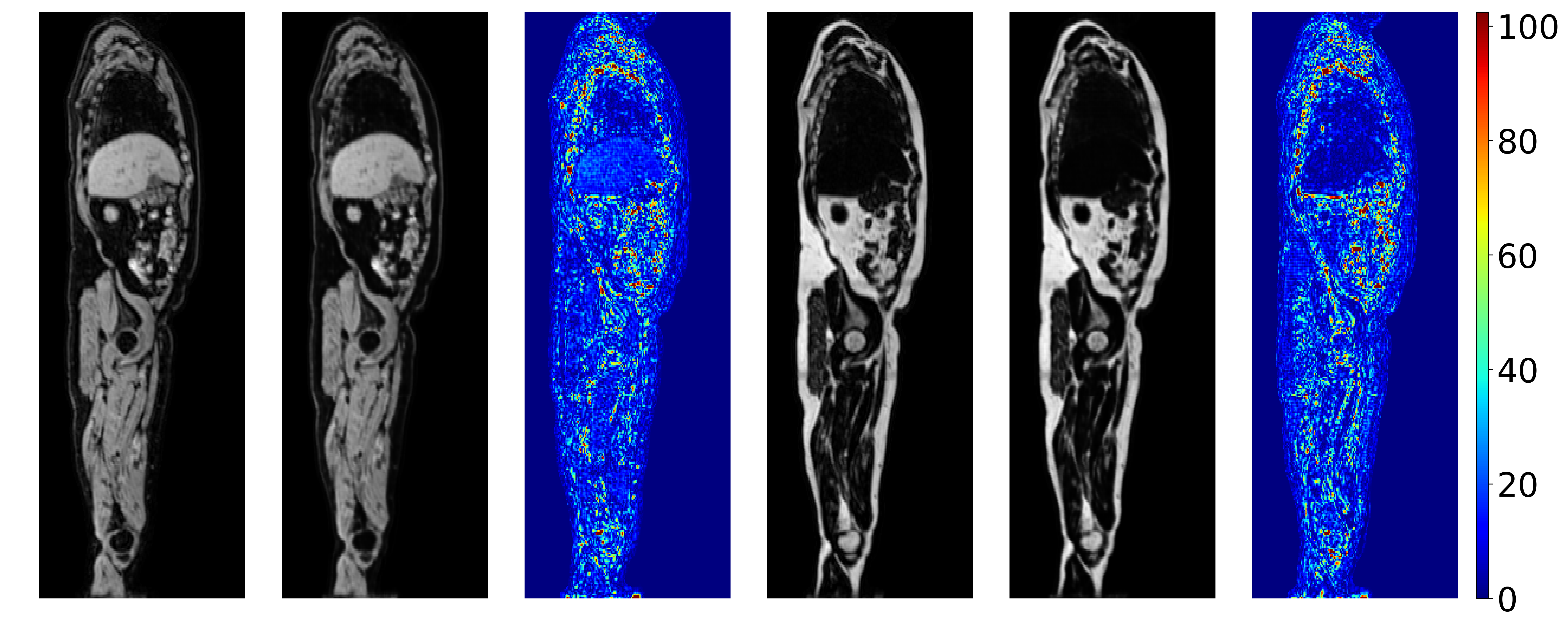

Supplement: Supplementary file 3 — Additional file 3. Original and predicted fat and water signals and their absolute differences in sagittal view with reduced dynamic range. [file 40537_2022_677_MOESM3_ESM.png]

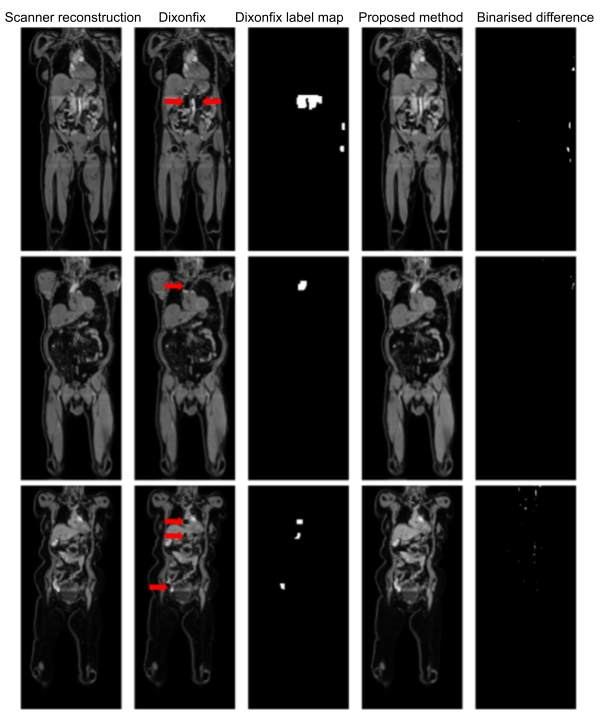

Supplement: Supplementary file 4 — Additional file 4. Comparative assessment of false positives between dixonfix and our method for four examples with swap-free original data. Red arrows point to false positive induced swaps. [file 40537_2022_677_MOESM4_ESM.png]

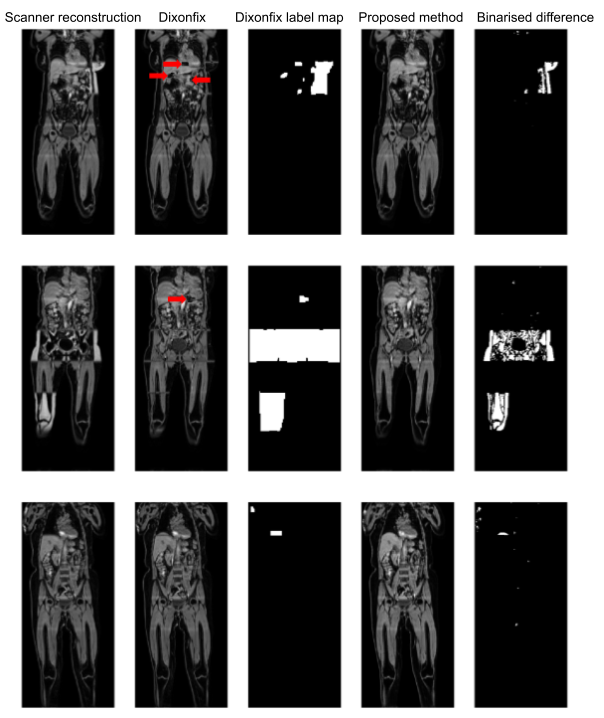

Supplement: Supplementary file 5 — Additional file 5. Comparative assessment of false positives between dixonfix and our method for four examples with original data containing swaps. Red arrows point to false positive induced swaps. [file 40537_2022_677_MOESM5_ESM.png]
